# An assessment of publishing practices and barriers faced by medical students to conduct research: A cross‐sectional study from Pakistan

**DOI:** 10.1002/hsr2.831

**Published:** 2022-09-11

**Authors:** Shoaib Ahmad, Shkaib Ahmad, Unaiza Ahmad, Huzaifa Ahmad Cheema, Nida Iqbal, Abia Shahid, Badar Malik, Amna Siddique, Huda Jaffar, Usman Ghani, Waqar Sarfraz, Vrushali Shelar, Ufaq Rahir, Maryam Zubair, Nazia Nikhat Ali, Sifwa Safdar, Mohammad Yasir Essar, Zain Ul Abadeen

**Affiliations:** ^1^ Department of General Medicine Punjab Medical College Faisalabad Pakistan; ^2^ Department of General Medicine DG Khan Medical College DG Khan Pakistan; ^3^ Department of General Medicine King Edward Medical University Lahore Pakistan; ^4^ Department of General Medicine Mohtarma Benazir Bhutto Shaheed Medical College Azad Jammu and Kashmir Pakistan; ^5^ Department of General Medicine Karachi Medical and Dental College Karachi Pakistan; ^6^ Department of General Medicine Dow Medical University Karachi Pakistan; ^7^ Department of General Medicine Saratov State Medical University Saratov Russia; ^8^ Department of General Medicine JSS Medical College Mysuru India; ^9^ Department of General Medicine University of Tripoli Tripoli Libya; ^10^ Department of General Medicine Allama Iqbal Medical College Lahore Pakistan; ^11^ Department of Dentistry Kabul University of Medical Sciences Kabul Afghanistan

**Keywords:** medical students, Pakistan, publishing practices and barriers

## Abstract

**Background:**

Pakistan being a low‐ and middle‐income country, its institutes are substantially deficient in scientific and technological aspects and share limited research contributions to the world repositories. Therefore, there is a rising concern to reflect on the history and status of publishing attitudes among medical students in Pakistan and to highlight and address the barriers that they are facing.

**Methods:**

A study was conducted aiming to determine the experience, motivation, and attitude of medical students in regarding publishing practices throughout Pakistan in several medical colleges. A multivariable logistic regression model was used to find the independent predictors of students publishing a research article. Forward selection was used to arrive at the final stepwise logistic regression. Odds ratio (OR) and 95% confidence interval (CI) were calculated. *p* < 0.05 was considered significant for all statistical tests.

**Results:**

From a sample size of 1225 participants, only 6.6% of students had published an article in our study. Of these, 59% were males and 31.3% were in final year. Males were more likely to publish articles than females (OR = 2.69, 95% CI: 1.37–5.26) and final‐year students were more likely to publish articles than first‐year students (OR = 7.48, 95% CI: 1.34–41.81). Students that had the knowledge that performing research is the way through which they will be judged for jobs had significantly higher odds of getting an article published (OR = 16.21, 95% CI: 3.65–71.88). Additionally, students who had been taught how to write a paper and those who knew the process of submitting an article were more likely to get published than the others.

**Conclusion:**

Our study has successfully highlighted the status of publishing among medical students in Pakistan. Our findings serve as an eye opener and call to action for authorities to address the grievances of students in terms of barriers, lack of mentorship, and lack of research teaching. We hope our findings can guide a strong policy change to facilitate the next generation of passionate researchers.

## 1 INTRODUCTION

Globally a growing trend has been observed in research on the creation and use of scholarly communication in all fields of study including medicine. Turning the focus around its pivotal role in improving health care.^1^ It also bolsters medical students' critical thinking skills, ability to explore literature, teamwork, and writing skills.^2^ Furthermore, performing research at an undergraduate level, not only maintain the student's attitude toward doing research throughout their course of study but also ease one's chance to make a career in the field of medicine.^3^ In addition to the latter publishing, is now a form of assessing career and self‐development to recruit the doctors in this era of amplified competition than before.^3^

Notably, the trustworthiness, respect, and ranking of higher education institutions (HEI) are primarily determined by their academic integrity and their status in publishing ethical scientific research. Hence, academic institutions from high‐income countries are continuously striving to enhance the quality of education and research to eventually nurture their ranking and prestige.^2^ As reported by the study of Kanwal Ameen, most of the world's scholarly communication is concentrated in a few HIC that are scientifically equipped with technologies such as, that is, United States, United Kingdom, and other European countries. On the contrary, HEI of low‐ and middle‐income countries (LMICs) is not only behind in deliverance of quality education but also in promoting research publishing.^4^

Considering, Pakistan, is the fifth most populous country in the world after China, India, the United States, and Indonesia.^5^ Further, its annual intake is about 15,000 medical students. Out of almost 3000 medical colleges in the world, there are around 114+ medical colleges in the state, with around 38% public and 62% private. At the provincial level, Sindh and Punjab, and the Federal area own around more than 50% of medical colleges remaining covered by other provinces of the state, that is, Baluchistan, Khyber Pakhtunkhwa (KPK), and Azad Jammu and Kashmir (AJK).^6^ Pakistan being an LMIC its HEIs are substantially deficient in scientific and technological aspects and share limited research contributions to the world repositories also.^7^ Therefore, there is a rising concern to reflect on the history and status of publishing attitudes among medical students in Pakistan. To understand where it stands today and what needs to be done to upgrade it. Thus, to the best of our knowledge, this study is the first to assess the publishing practices of Pakistan medical students to investigate potential barriers to performing research and submitting papers.

## 2 METHODS

A study was conducted aiming to determine the experience, motivation, and attitude of medical students regarding publishing practices. This study was conducted throughout Pakistan in several medical colleges. Any medical student falling under the age group between 18 and 30 years was eligible to participate in the study. No specific gender or ethnic group was given significance.

Ethical approval was taken from Faisalabad Medical University, Faisalabad. Informed consent for voluntary participation was taken from the participants after declaring the study objectives at the start of the questionnaire. Respondent anonymity and confidentiality were insured by design. The guidelines outlined in the Helsinki declaration were followed in this study.

To collect data, an online questionnaire on Google Forms was circulated among medical students across Pakistan through social media. The questionnaire was previously constructed and used by other researchers (3) and was modified to suit our objectives. The validity of the questionnaire was checked by Cronbach alpha which was greater than 0.7. The first section of the questionnaire was about basic demographics of the participant. The second section was based on experiences in the field of research and contained questions regarding motivation behind writing a paper and factors contributing to choosing a journal for publication. The third and fourth sections were more specifically related to past expertise in research and attitudes toward publishing.

Analysis was conducted using SPSS version 26.0 (IBM SPSS Statistics). Descriptive statistics were calculated as frequency and percentages for categorical variables, and means with standard deviation (SD) for continuous variables included in our study. Associations between variables were explored by the Pearson's Chi‐Square test. Chi‐Square continuity correction was applied where appropriate. A multivariable logistic regression model was used to find the independent predictors of students publishing a research article. Forward selection was used to arrive at the final stepwise logistic regression. A variable was only included in the model if it improved the model which was checked by the Akaike information criterion (2), and if the Omnibus likelihood ratio test for it was significant. Odds ratio (OR) and 95% confidence interval (CI) were calculated. *p* < 0.05 was considered significant for all statistical tests.

## 3 RESULTS

### 3.1 Participants and descriptive statistics

A total of 1252 students from medical schools all over Pakistan participated in our study. The mean age of the participants was 21.2 years (SD: 1.65). The majority were female (61.3%) and were from the province of Punjab (85.7%). The year of study varied among the students (Table 1).

**Table 1:** Descriptive statistics of participants

Variables		Number of students out of all participants (%)	Number of students out of those who published (%)
Age (mean ± SD)		21.16 ± 1.65	21.75 ± 1.51
Gender	Male	484 (38.7)	49 (59.0)
	Female	768 (61.3)	34 (41.0)
Province	Balochistan	20 (1.6)	0 (0.0)
	KPK	24 (1.9)	0 (0.0)
	Punjab	1073 (85.7)	46 (55.4)
	Sindh	135 (10.8)	37 (44.6)
Year of Education	First year	151 (12.1)	2 (2.4)
	Second year	171 (13.7)	8 (9.6)
	Third year	445 (35.5)	23 (27.7)
	Fourth year	397 (31.7)	23 (27.7)
	Final year	80 (6.4)	26 (31.3)
	Graduate	8 (0.6)	1 (1.2)
Article Published	Yes	83 (6.6)	‐
	No	1169 (93.4)	‐

### 3.2 Publishing status of students

**Figure 1:**
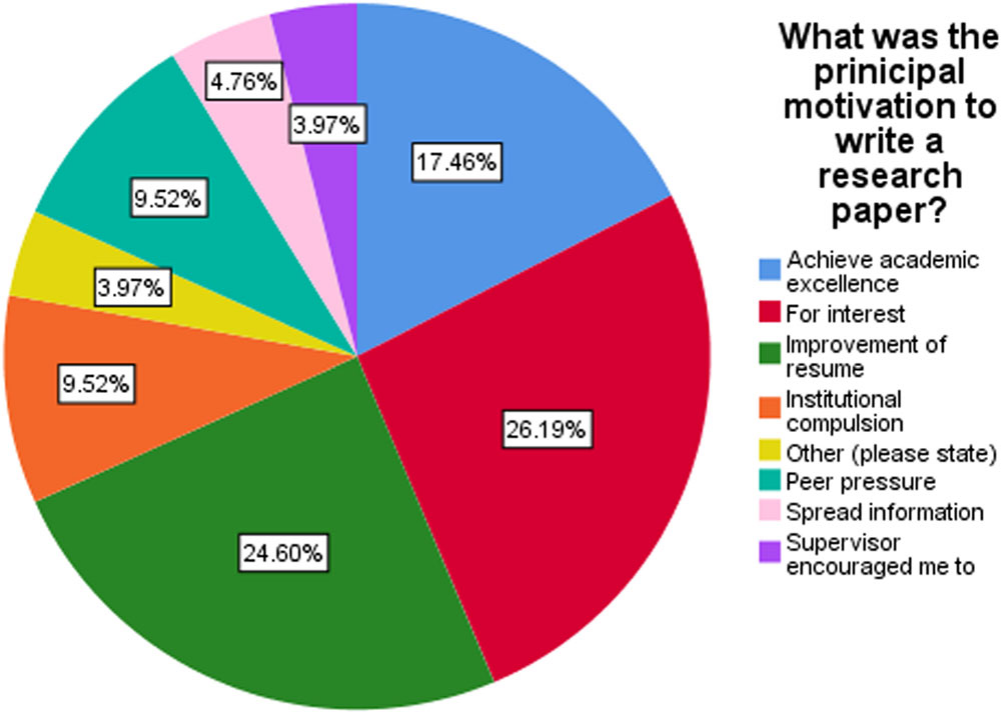
Pie chart of what was the principal motivation to write a research paper

**Figure 2:**
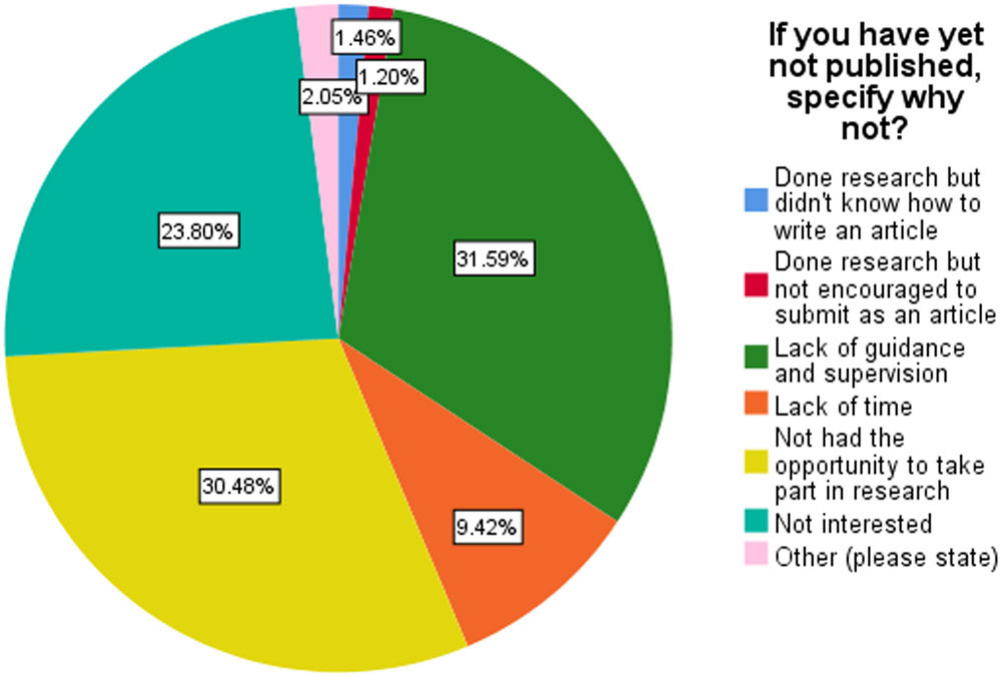
Pie chart of reasons for not getting an article published

Only 6.6% of students had published an article in our study. Of these, 59% were males and 31.3% were in final year (Table 1). Males (10.1%) were more likely to publish an article than females (4.4%; Pearson's Chi‐Square test, *p* < 0.001). Year of study was also significantly associated with whether students published articles with final year students publishing more articles (32.5%; Pearson's Chi‐Square test, *p* < 0.001). Of the students that had published articles, the type of article, their author rank, and the outcome of their submission varied (Table 2). The principal motivation behind publishing articles was highlighted as improvement of resume (24.6%) and interest (26.2%; Figure 1). When asked which factor affected their choice of journal the most, 32.2% said it was the journal repute while 23.5% said it was the possibility of acceptance. The main hindrances to not publishing an article were stated as lack of guidance and supervision (31.6%), not having the opportunity to take part in research (30.5%), and being not interested (23.8%) by the students (Figure 2).

**Table 2:** Types of articles published and outcomes of submission

	First author	Second author	Third author	Fourth author	Other author
Original paper	21	22	16	3	11
Review	16	30	8	4	9
Case report	22	11	16	1	8
Letter	23	17	18	2	10
Abstract	9	21	13	5	8
Other	14	24	8	3	10

	**Accepted with revision**	**Accepted without revision**	**Rejected outright**	**Revision in progress**
Original paper	49	12	3	9
Review	45	17	2	6
Case report	37	9	0	0
Letter	27	33	2	4
Abstract	28	15	0	7
Other	26	16	3	8

### 3.3 Research participation of the students

The vast majority of the students (70.4%) had not taken part in either a research project or an audit and only 25.9% and 1.7% had taken part in a research project and an audit, respectively; of these, 90.6% had taken part in less than 5 research projects or audits. Most of these projects were questionnaire‐based (64.1%). Of the students that had taken part in research, 53.9% stated that they had conducted projects in the career they wished to pursue.

### 3.4 Teaching and opportunity to take part in research

Majority of the students said that they would like more opportunity to take part in research or audit projects (73.4%). Only 9.0% wanted to take part in laboratory research while the rest indicated that they would like to participate in clinical research. Most of the students felt that they had not been encouraged by their seniors to get involved in research (66.0%). A large proportion of students wanted to take part in research in any area just to gain experience (40.6%) while 34.9% only wanted to take part in their specialty of interest. Eighty percent of the students had never applied for ethical approval for a research project.

### 3.5 Journal reading, presentations, and posters

Fifty‐seven percent of the students read journal articles with most of them stating that it was either for interest (35.9%) or to improve their knowledge (48.5%). The major reasons for not reading journal articles were not being encouraged to do so (33.7%) and not being interested (31.5%). Only 10.1% had submitted an abstract to a scientific conference. Forty‐six percent had presented a poster or given an oral presentation at a conference. Only 36.7% of the medical students said that they were encouraged by their seniors to get involved in presenting at a medical conference.

### 3.6 Teaching received on writing papers

Only 18% of the students said that they knew how to critique a paper. Thirty percent of the students felt that they could write an abstract while 60.4% felt that they knew how to write a paper. Only 17.7% and 26.4% of the students had been taught how to write an abstract and paper, respectively. Regarding the publishing process, 19.8% stated that they knew how to submit an article while only 25.0% were confident of submitting an article without supervision. Thirty‐five percent of the students wanted to be taught how to write an abstract, 40.5% for a paper and 51.1% wanted teaching on publishing practices. The majority of the students felt it was important to publish a paper (73.4%) with the main reasons being to improve their career (41.7%) and it is an important skill to learn (34.1%).

### 3.7 Knowledge in the importance of publishing

Overall, 60.2% of the students knew that they are expected to have taken part in research projects during their time at the medical school. A similar proportion of students (59.1%) said that they knew that submitting papers and performing research is the way through which they will be judged for jobs later in their careers. Fifty‐eight percent agreed that the survey has convinced them to begin seeking opportunities to perform research or audits.

### 3.8 Barriers to publishing

Stepwise logistic regression model showed that males were more likely to publish articles than females (OR = 2.69, 95% CI: 1.37–5.26) and final‐year students were more likely to publish articles than first‐year students (OR = 7.48, 95% CI: 1.34–41.81). Students that had the knowledge that performing research is the way through which they will be judged for jobs had significantly higher odds of getting an article published (OR = 16.21, 95% CI: 3.65–71.88). Additionally, students who had been taught how to write a paper and those who knew the process of submitting an article were more likely to get published than the others (Table 3).

**Table 3:** Independent predictors of getting an article published

Predictor		OR	95% CI		*P*‐value
			Lower	Upper	
Gender	Female	Referent	‐	‐	‐
	Male	2.687	1.372	5.26219	0.004
Medical college year	First year	Referent	‐	‐	‐
	Second year	1.887	0.267	13.31783	0.524
	Third year	3.301	0.652	16.71418	0.149
	Fourth year	4.354	0.864	21.93942	0.075
	Final year	7.482	1.339	41.81171	0.022
Do you know that submitting papers and performing research is the way in which you are judged for jobs later in your career?	No	Referent	‐	‐	‐
	Yes	16.206	3.654	71.88005	<0.001
Do you know you will be expected to have performed audits, started to submit papers during your professional years at medical college?	No	Referent	‐	‐	‐
	Yes	0.967	0.411	2.27938	0.939
Have you been taught how to write an abstract?	No	Referent	‐	‐	‐
	Yes	2.019	0.914	4.45993	0.082
Have you been taught how to write a paper?	No	Referent	‐	‐	‐
	Yes	4.146	1.861	9.23461	<0.001
Are you encouraged by your seniors to get involved in research/audit work?	No	Referent	‐	‐	‐
	Yes	1.033	0.485	2.20353	0.932
Do you feel you know the process of submitting an article?	No	Referent	‐	‐	‐
	Yes	3.899	1.801	8.43977	<0.001
Would you feel confident in submitting an article, without supervision?	No	Referent	‐	‐	‐
	Yes	1.645	0.814	3.32294	0.166

Abbreviations: CI, confidence interval; OR, odds ratio.

## 4 DISCUSSION

In our study of 1252 participants, we found that only 6.6% of students had published an article in our study which is an alarmingly low percentage. Our reported percentage was less than that of previous studies conducted in both HICs; Britain (14%),^3^ Sweden (15%),^8^ LMICs India (17.4%),^9^ Uganda (22.5%),^10^ and Nigeria (34.3%).^11^ We found gender disparities in the publishing practices; males formed 59% of students who had an article published. We found that being from the male gender made it more likely 10.1% versus females 4.4% to get articles published. These gender disparities are echoed throughout the literature.^10^, ^12^, ^13^, ^14^ There can be multiple reasons for why this gender gap exists. First, Pakistan has a conservative religious society with limited opportunities for women in education and career progression although trends are gradually changing. Studies in Saudi Arabia, a similar conservative setting also reports gender‐specific barriers and attributes this to a lack of access to patients/samples and a scarcity of same‐sex mentors/role models^15^, ^16^ Other factors known to perpetuate the gender gap include; sexism in the research environment (including inequity in resource distribution and remuneration, etc.), deep‐seated concepts of traditional gender‐roles, and differences in career preference (e.g., choosing clinical practice over academia).^17^ Educationalists need to address these gender imbalances by having a targeted approach toward women; providing research incentives to increase participation and increasing the availability of gender‐specific mentorship programs.^18^

We found a strong association between the year of study and likelihood of publishing an article with final year students publishing more articles (32.5%). This trend of publishing status improving with year of study and age is also reflected across the published data^3^, ^10^, ^19^ with a British study reporting 70% of the participants who had published were either in fifth year or doing an intercalated degree.^3^ This can be a result of increased research courses and educational opportunities in the later years either integrated in curriculum or independent, overtime students are likely to have been exposed to research networks, formed collaborations with peer and senior researchers.^10^ By final years students are likely to have aligned their career priorities and maybe better able to invest and manage their time in research activities.

When inquired about the key motivating factors students highlighted improvement of resume 24.6% and personal interest 26.2% as their driving force. These findings match with those in Uganda,^10^ Nigeria,^11^ and Columbia^19^ where participants reported interest in publishing and curiosity as well as having strong role models, personal development, and contribution to patient care. Pallampathy et al.^20^ and Indian^9^ studies also reported personal interest but also mentioned factors that contrasted with our findings such as facilitation of foreign exams, and peer pressure as their main motivational factors for research involvement. Improving resumes is an important factor especially in the context of applying to residency^21^ Indeed engaging in research opportunities is known to result in improved soft skills such as teamwork, leadership, communication, and so on and also increases the chances of getting admission into postgraduate courses.^22^

Reflecting on the student inclinations toward journals the majority said 32.2% chose the journal based on journal reputation and 23.5% said based on possibility of acceptance. Studies in LMICs have found students preferring internationally reputable journals due to chances of submission fee waivers, and the added visibility and acknowledgment.^10^ Additionally, students fear rejection since journals might prefer articles from experts in the field therefore, it is imperative that high‐impact journals should create sections reserved only for students to avoid discrimination based on education level of the author.^23^

The main hindrances to not publishing an article were stated as lack of guidance and supervision (31.6%), not having the opportunity to take part in research (30.5%), and being not interested (23.8%) by the students. Indeed, lack of supervision and mentorship has been repeatedly highlighted in Pakistan^2^, ^13^, ^24^ and others.^15^, ^16^ Also reported in these studies was lack of time and funding. We didn't find lack of finances as the main barrier and this gives an interesting insight into our research population. It is possible that most of them have not conducted research previously and therefore request mentorship and education as their primary needs. In contrast, those who have published previously may have faced financial challenges in carrying out and publishing research in journals and therefore well aware of the role of funding in facilitating research.^13^ The impact of these barriers is reflected in the fact that the vast majority of our participants, 70.4%, had not taken part in either a research project or an audit. Questionnaire‐based research format was popular (64.1%) among those who conducted research potentially due to its advantage of less time‐consuming nature and being relatively easy to perform. Furthermore, students lack the opportunities to get involved in research and expressed their desire to have more opportunities 73.4%. Only 9% of the participants showed an inclination toward lab‐based research opportunities compared to the rest favoring clinical research a finding congruent with the study conducted by Stockfelt et al.^25^ who found that only 1/10th were interested in basic science research Teaching faculty and researchers should encourage students to work alongside them to improve medical students' exposure to these formats. Most of the students felt they weren't encouraged by their seniors to get involved in research. A prevalent research culture and peer support networks can serve as role models and inspiration for students in earlier years. A continuous flow of guidance and mentorship is necessary to prevent passionate students from losing interest.^19^ Additionally, a majority of students stated that they are willing to be engaged in any research project regardless of their specialty of interest just to gain experience. This sentiment pinpoints the underlying situation of rarity of opportunities in general and a self‐realization on part of the students that once they have developed the necessary skill set more doors of opportunities will open for them. As Habineza et al.^26^ showed in a research conducted in Rwanda the main advantage felt by students involved in research is learning how to conduct research.

Trends in journal readership have improved 57% as compared to past years 20%^2^ but are still low compared to studies in Britain which reported 78% of students reading journal articles.^3^ Factors that favored reading journal articles included improving knowledge and for interest. On the other hand, discouraging factors included not being encouraged by seniors and lack of interest. It should be emphasized that a habit of reading articles not only is fruitful in documenting one's own research but keeps one updated about the new scientific advances in the field, which forms the basis of good, evidence‐based clinical practice. Regarding abstract/poster presentation to scientific conferences only 10.1% had submitted an abstract and 47% presented a poster or given an oral presentation at a conference, indicating again a lack of encouragement by seniors as a limiting factor. These findings are similar to Griffin et al. 17% had submitted an article for scientific meetings. These findings highlight the lack of awareness among medical students about the benefits of presenting their work in conferences. Another reason might be the minimal role of students in authorship of the paper, where their task is mostly limited to data collection and analysis and hence not considered serving/qualified to present the paper. Additionally, the impact of COVID‐19 pandemic cannot be discounted which resulted in the cessation of all in‐person conferences even though Zoom meetings with its worldwide outreach was a viable alternative. Poster presentation is a great opportunity to get involved in conferences, which is a great opportunity to practice and improve one's communication skills but also form networks and collaborations by showcasing one's work to other people in the field.

Another crucial finding in our study was the huge deficit in teaching received by students on writing research papers. Only 18% of our students knew how to critique a paper this is in contrast to Griffin et al.^3^ who reported 49% of students being able to critique. Components should be introduced in the medical curriculum that teach students this critical skill. In an era of highly accessible information, this equips medical students with tools to filter quality information from misinformation to make informed decisions about their patients' care. Similarly, only 30% of students were confident in their ability to write an abstract and 60.4% on writing a paper which is unsurprising considering only 17.7% and 26.4% of the students had been taught how to write an abstract and paper, respectively. Additionally, we found that students who had been taught how to write a paper and those who knew the process of submitting an article were more likely to get published than the others. Further, only a minority of students knew how to submit an article or submit it without supervision Hence, there is an urgent need to introduce research methodology courses and workshops tailored to teach students about writing abstracts, papers, and guide them about publishing practices so they can confidently embark on their research journeys. It should be emphasized that our findings sharply contrast with that of a study conducted in Aga Khan University (AKU) in Pakistan.^27^ They claimed that 90% of AKU medical students felt confident in understanding and writing a research paper and 28.4% claiming their ability to do so without assistance. It should be stressed that the curriculum of AKU is different from what is practiced in the rest of the country. Their medical curriculum in developing research skills among medical students through well‐structured intensive training. Their students are taught theoretical essentials of research methodology, statistics, and epidemiology during the first 2 years of their medical school, followed by rigorous student‐led community health projects during years 4 and 5.^27^

Regarding the perceived knowledge about publishing, 60.2% of the students knew that they are expected to have taken part in research projects during their time at the medical school. A similar proportion of students (59.1%) said that they knew that submitting papers and performing research is the way through which they will be judged for jobs later in their careers, a finding consistent with previous studies.^3^ Hence, it can be deduced that not everyone is aware of the importance of publishing and therefore would be interested in publishing. Further, we found that students that had the knowledge that performing research is the way through which they will be judged for jobs had significantly higher odds of getting an article published. With residency matches becoming increasingly competitive both home and abroad, the number of publications to an applicant's credit helps them stand out to the evaluator. Medical Students would benefit greatly if universities arranged seminars/lectures and invited renowned researchers to deliver talks to the student about the importance of publishing.

To ameliorate the current publishing situation among medical students in Pakistan, we have a few suggestions that can serve as a blueprint for educationist and concerned authorities to reform policies and introduce interventions. First, we emphasize the need for strong mentorship; supervisors and faculty in the fields of student interest that are easily approachable and can be consulted for queries. Efforts should be made to launch same‐gender mentorship programs that are culturally competent to bridge the gender gap. Second, curriculum reforms that include mandatory research, integrated throughout the 5 years of medical curriculum not limited to the final years should be introduced. These reforms should be targeted to address the various deficiencies in student knowledge as regards to research methodology especially the writing and publishing process. Third, universities should actively incentivize students to participate in research projects by announcing funding grants, scholarships, waivers and journal fee sponsors, research competitions, and curriculum credit points. Fourth, opportunities should be created for students to contribute to research in an impactful way beyond the middle author role. Senior members of the faculty can play their role here. Efforts should be made to enhance collaborations locally, regionally, and internationally; universities can sign MOUs and contracts that allow their students to go on research exchange programs from LMICs to HICs and vice versa so that there is an equitable sharing of knowledge and resources. Finally, “peer‐led research societies” and “student interest groups”^28^ should be made so that students with similar career/research interests can freely learn and discuss their queries. These can act as safe spaces where seniors and juniors can collaborate and initiate mutually beneficial activities such as research capacity building and literacy, and as a media to announce and disseminate new research opportunities.

Our study is the largest and most recent study to look at publishing practices across Pakistan. We are confident about the accuracy and generalizability of our results because the majority of our responses were from Punjab and Sindh, two provinces with the highest density of medical colleges in Pakistan. However, our study might have some limitations. First, by the nature of study design, our study is a cross‐sectional study which is a snapshot in time and therefore not possible to analyze any trends in the publishing practices over the years. Second, it is an observational study and respondents couldn't respond in free text, a future study with this addition can fully encapsulate the sentiments of the students. Third, we relied on students telling us the correct information about their publication status and didn't employ any method to check the accuracy of their claim.

## 5 CONCLUSIONS

Our study has successfully highlighted the status of publishing among medical students in Pakistan. Our findings serve as an eye opener and call to action for authorities to address the grievances of students in terms of barriers, lack of mentorship, and lack of research teaching. We hope our findings can guide a strong policy change to facilitate the next generation of passionate researchers.

## AUTHOR CONTRIBUTIONS

**Shoaib Ahmad**: Conceptualization; Supervision; Writing – original draft. **Shkaib Ahmad**: Writing – original draft. **Unaiza Ahmad**: Writing – original draft. **Huzaifa Ahmad Cheema**: Formal analysis; Methodology. **Nida Iqbal**: Data curation. **Abia Shahid**: Formal analysis; Methodology. **Badar Malik**: Data curation. **Amna Siddique**: Writing – original draft. **Huda Jaffar**: Data curation. **Usman Ghani**: Data curation. **Waqar Sarfraz**: Data curation. **Vrushali Shelar**: Writing – review & editing. **Ufaq Rahir**: Writing – review & editing. **Maryam Zubair**: Writing – review & editing. **Nazia Nikhat Ali**: Writing – review & editing. **Sifwa Safdar**: Writing – original draft. **Mohammad Yasir Essar**: Conceptualization. **Zain Ul Abadeen**: Conceptualization; Writing – review & editing.

## CONFLICT OF INTEREST

The authors declare no conflict of interest.

## TRANSPARENCY STATEMENT

The lead author Mohammad Yasir Essar affirms that this manuscript is an honest, accurate, and transparent account of the study being reported; that no important aspects of the study have been omitted; and that any discrepancies from the study as planned (and, if relevant, registered) have been explained.

## Data Availability

All data are available in the article.

## References

[1] Srinivasan M , Poorni S , Kumar Sn , Sujatha G . Research experiences, attitudes, and barriers to publishing among the dental postgraduate teachers: a cross‐sectional study. Indian J Dent Res. 2014;25(4):454‐458. 10.4103/0970-9290.142529 25307908

[2] Aslam F , Shakir M , Qayyum MA . Why medical students are crucial to the future of research in South Asia. PLoS Med. 2005;2(11):e322. 10.1371/journal.pmed.0020322 16288553 PMC1297528

[3] Griffin MF , Hindocha S . Publication practices of medical students at British medical schools: experience, attitudes and barriers to publish. Med Teach. 2010;33(1):e1‐e8. 10.3109/0142159x.2011.530320 21182368

[4] Ameen K . Practices of quality and trustworthiness in scholarly communication: a case from Pakistan. Learn Publ. 2017;30(2):133‐142. 10.1002/leap.1094

[5] Qureshi Z . Pakistan surpasses Brazil to become world's 5th most populous country. Gulf News. Published July 12, 2020. Accessed June 20, 2022. https://gulfnews.com/world/asia/pakistan/pakistan-surpasses-brazil-to-become-worlds-5th-most-populous-country-1.72557051

[6] Bokhari DW . Medical colleges and doctors in Pakistan—too many or too few? Latest News—The Nation. Published September 21, 2019. Accessed June 20, 2022. https://nation.com.pk/2019/09/21/medical-colleges-and-doctors-in-pakistan-too-many-or-too-few/

[7] Saiyid A . History of publishing. TNS. Published November 28, 2021. Accessed June 20, 2022. https://www.thenews.com.pk/tns/detail/911903-pakistans-publishing-industry

[8] Möller R , Shoshan M . Medical students' research productivity and career preferences; a 2‐year prospective follow‐up study. BMC Med Educ. 2017;17(1):51. 10.1186/s12909-017-0890-7 28253880 PMC5335804

[9] Manoharan A , Chellaiyan V , Jasmine M , Liaquathali F . Medical research: perception and barriers to its practice among medical school students of Chennai. J Educ Health Promot. 2019;8(1):134. 10.4103/jehp.jehp_464_18 31463319 PMC6691744

[10] Kiyimba B , Atulinda L , Nalunkuma R , et al. Research involvement among undergraduate health profession students in a resource‐limited setting: awareness, attitude, motivators and barriers. BMC Med Educ. 2022;22(1):249. 10.1186/s12909-022-03320-y 35387633 PMC8985566

[11] Iloh GP , Onya O , Nwamoh U , Onyemachi PN , Chukwuonye M , Godswill‐Uko E . Patient–doctor relationship in underserved environment: a cross‐sectional study of attitudinal orientation, practice inclination, barriers and benefits among medical practitioners in Abia State, Nigeria. Niger Postgrad Med J. 2019;26(2):87‐93. 10.4103/npmj.npmj_13_19 31187747

[12] Bovijn J , Kajee N , Esterhuizen TM , Van Schalkwyk SC . Research involvement among undergraduate health sciences students: a cross‐sectional study. BMC Med Educ. 2017;17(1):186. 10.1186/s12909-017-1025-x 29037185 PMC5644181

[13] Mahmood Shah SM , Sohail M , Ahmad KM , Imtiaz F , Iftikhar S . Grooming future physician‐scientists: evaluating the impact of research motivations, practices, and perceived barriers towards the uptake of an academic career among medical students. Cureus. 2017;9:1991. 10.7759/cureus.1991 PMC5828671 29503785

[14] Mugabo E , Velin L , Nduwayezu R . Exploring factors associated with research involvement of undergraduate students at the College of Medicine and Health Sciences, University of Rwanda. BMC Med Educ. 2021;21(1):239. 10.1186/s12909-021-02662-3 33902555 PMC8072743

[15] Noorelahi M , Soubhanneyaz A , Kasim K . Perceptions, barriers, and practices of medical research among students at Taibah College of Medicine, Madinah, Saudi Arabia. Adv Med Educ Pract. 2015;6:479‐485. 10.2147/amep.s83978 26185479 PMC4500619

[16] Kharraz R , Hamadah R , AlFawaz D , et al. Perceived barriers towards participation in undergraduate research activities among medical students at Alfaisal University—College of Medicine: a Saudi Arabian perspective. Med Teach. 2016;38(suppl 1):S12‐S18. 10.3109/0142159x.2016.1142507 26984028

[17] Jagsi R , Guancial EA , Worobey CC , et al. “Gender gap” in authorship of academic medical literature—a 35‐year perspective. N Engl J Med. 2006;355(3):281‐287. 10.1056/nejmsa053910 16855268

[18] Farkas AH , Bonifacino E , Turner R , Tilstra SA , Corbelli JA . Mentorship of women in academic medicine: a systematic review. J Gen Intern Med. 2019;34(7):1322‐1329. 10.1007/s11606-019-04955-2 31037545 PMC6614283

[19] Bonilla‐Escobar FJ , Bonilla‐Velez J , Tobón‐García D , Ángel‐Isaza AM . Medical student researchers in Colombia and associated factors with publication: a cross‐sectional study. BMC Med Educ. 2017;17(1):254. 10.1186/s12909-017-1087-9 29246229 PMC5732498

[20] Basavareddy A , Pallamparthy S . Knowledge, attitude, practice, and barriers toward research among medical students: a cross‐sectional questionnaire‐based survey. Perspect Clin Res. 2019;10(2):73‐78. 10.4103/picr.picr_1_18 31008073 PMC6463502

[21] Pathipati AS , Taleghani N . Research in medical school: a survey evaluating why medical students take research years. Cureus. 2016;8:741. 10.7759/cureus.741 PMC5026499 27672532

[22] Amorim FF , Santana LA , Toledo IL , et al. Undergraduate research in medical education. Rev Assoc Med Bras. 2017;63(12):1017‐1018. 10.1590/1806-9282.63.12.1017 29489981

[23] Llamas‐Nieves A , Maiguel‐Lapeira J , Lozada‐Martinez I , Torres‐Llinas D , Moscote‐Salazar L . The desire to publish a scientific article and the difficulties of publishing it in a high‐quality neurosurgery scientific journal. J Neurosurg Sci. 2022;66(2):163‐164. 10.23736/s0390-5616.21.05297-8 33709666

[24] Kumar J , Memon A , Kumar A , Kumari R , Kumar B , Fareed S . Barriers experienced by medical students in conducting research at undergraduate level. Cureus. 2019;11:4452. 10.7759/cureus.4452 PMC6561510 31205838

[25] Stockfelt M , Karlsson L , Finizia C . Research interest and activity among medical students in Gothenburg, Sweden, a cross‐sectional study. BMC Med Educ. 2016;16(1):226. 10.1186/s12909-016-0749-3 27565878 PMC5002212

[26] Habineza H , Nsanzabaganwa C , Nyirimanzi N , et al. Perceived attitudes of the importance and barriers to research amongst Rwandan interns and pediatric residents—a cross‐sectional study. BMC Med Educ. 2019;19(1):4. 10.1186/s12909-018-1425-6 30606184 PMC6318911

[27] Khan H , Khawaja MR , Waheed A , Rauf MA , Fatmi Z . Knowledge and attitudes about health research amongst a group of Pakistani medical students. BMC Med Educ. 2006;6(1):54. 10.1186/1472-6920-6-54 17081286 PMC1635552

[28] Miranda‐Pacheco JA , De Santis‐Tamara SA , Parra‐Pinzón SL , González‐Monterroza JJ , Lozada‐Martínez ID . Medical interest groups and work policies as emerging determinants of a successful career: a student perspective—correspondence. Int J Surg. 2021;92:106020. 10.1016/j.ijsu.2021.106020 34252595

